# Solution-Processed Gallium–Tin-Based Oxide Semiconductors for Thin-Film Transistors

**DOI:** 10.3390/ma11010046

**Published:** 2017-12-28

**Authors:** Xue Zhang, Hyeonju Lee, Jungwon Kim, Eui-Jik Kim, Jaehoon Park

**Affiliations:** 1 Department of Electronic Engineering, Hallym University, Chuncheon 24252, Korea; zhangxue00@naver.com (X.Z.); zoozs123@naver.com (H.L.); 2 Department of Environmental Sciences & Biotechnology, Hallym University, Chuncheon 24252, Korea; jwkim@hallym.ac.kr; 3 Department of Convergence Software, Hallym University, Chuncheon 24252, Korea; ejkim32@hallym.ac.kr

**Keywords:** oxide semiconductor, sol-gel precursor, gallium, tin, transistor

## Abstract

We investigated the effects of gallium (Ga) and tin (Sn) compositions on the structural and chemical properties of Ga–Sn-mixed (Ga:Sn) oxide films and the electrical properties of Ga:Sn oxide thin-film transistors (TFTs). The thermogravimetric analysis results indicate that solution-processed oxide films can be produced via thermal annealing at 500 °C. The oxygen deficiency ratio in the Ga:Sn oxide film increased from 0.18 (Ga oxide) and 0.30 (Sn oxide) to 0.36, while the X-ray diffraction peaks corresponding to Sn oxide significantly reduced. The Ga:Sn oxide film exhibited smaller grains compared to the nanocrystalline Sn oxide film, while the Ga oxide film exhibited an amorphous morphology. We found that the electrical properties of TFTs significantly improve by mixing Ga and Sn. Here, the optimum weight ratio of the constituents in the mixture of Ga and Sn precursor sols was determined to be 1.0:0.9 (Ga precursor sol:Sn precursor sol) for application in the solution-processed Ga:Sn oxide TFTs. In addition, when the Ga(1.0):Sn(0.9) oxide film was thermally annealed at 900 °C, the field-effect mobility of the TFT was notably enhanced from 0.02 to 1.03 cm^2^/Vs. Therefore, the mixing concentration ratio and annealing temperature are crucial for the chemical and morphological properties of solution-processed Ga:Sn oxide films and for the TFT performance.

## 1. Introduction

Over the past few decades, metal-oxide semiconductors have attracted considerable attention owing to their thermal stability, wide band gap, high transmittance in visible light, and high electrical conductivity [1,2]. Studies show that the average transmittance of metal oxide films in the visible light region is greater than 87% and the electrical conductivity can be increased to 10^3^ S/cm [3,4]. Thus, the metal-oxide-based thin-film transistors (TFTs) are considered promising candidates for electronic applications, such as electronic memory devices, sensors, and flat panel displays [5,6,7].

To optimize the performance of metal-oxide-based TFTs, ternary and multicomponent oxides have been used as functional semiconductor layers, such as indium–zinc oxide, zinc–tin oxide, and indium–gallium–zinc oxide [8,9,10,11]. However, indium-based ternary and multicomponent oxide materials are associated with high manufacturing costs, because indium is a rare metal with few mining locations and is therefore expensive. It is well known that tin (Sn) and indium have similar electronic and electrical properties, and Sn is a relatively common metal. Thus, Sn has been widely studied as a material that can replace indium. Among the Sn-based multicomponent oxide materials, Ga is best suited to be mixed with Sn because the ionic radius of Ga^3+^ (62 pm) is close to that of Sn^4+^ (69 pm) [12,13]. This implies that the lattice distortion in the Ga–Sn-mixed (Ga:Sn) composite oxide films will be less significant than that for other compositions. However, the chemical bonds formed by Ga^3+^ ions with O^2−^ ions are weaker than those formed between Sn^4+^ and O^2−^ ions, i.e., Ga–O bonds are relatively weaker than Sn–O [14]. Accordingly, Ga:Sn oxide materials are expected to be utilized in electronic devices because the electrical properties of these materials can be modulated by the material composition ratio. Nevertheless, Ga:Sn oxide materials have not yet been studied for application in TFTs.

Tin oxide-based thin films can be prepared using various methods such as spray pyrolysis, sol-gel method, molecular beam epitaxy, plasma-enhanced atomic layer deposition, metal organic chemical vapor deposition (MOCVD), and direct current (DC) magnetron sputtering. Mi et al. [3] and Dang et al. [15] prepared Ga–Sn-mixed oxide thin films using MOCVD and DC magnetron sputtering methods, respectively. They reported that the carrier concentration was higher than 10^18^ cm^−3^. However, vacuum deposition methods require expensive equipment and therefore are associated with high manufacturing costs. We point out here that the sol-gel method can be processed under atmospheric pressure and that solution-based film coating can be performed in large areas.

In this study, we fabricated Ga oxide, Sn oxide, and Ga:Sn oxide thin films using the sol-gel solution process under ambient air conditions with gallium nitrate hydrate and tin chloride as precursors. The prepared sols and films were analyzed by thermogravimetry, X-ray photoemission spectroscopy (XPS), X-ray diffraction (XRD), and field-emission scanning electron microscopy (FE-SEM). The performances of the TFTs, in which Ga oxide, Sn oxide, and Ga:Sn oxide layers were used as a semiconductor, were investigated by analyzing their output and transfer characteristics. Experimental results demonstrate the significant impact of the material composition and thermal annealing temperature of Ga:Sn oxide semiconductor films on the TFT performance.

## 2. Experimental

**Figure 1:**
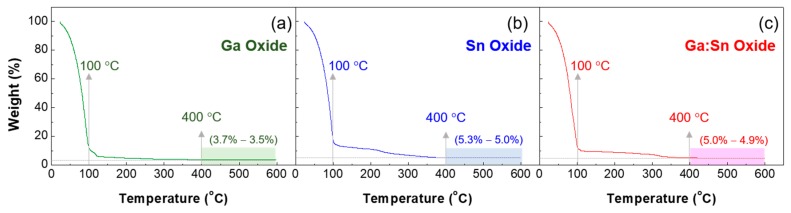
TGA (thermogravimetric analyzer) characteristic curves of the prepared sols: (**a**) the Ga oxide and (**b**) Sn oxide precursor solutions and (**c**) the mixture of Ga oxide and Sn oxide precursor solutions.

Gallium nitrate hydrate (Ga(NO_3_)_3_·xH_2_O) (255.74 g/mol, Sigma-Aldrich, St. Louis, MO, USA) and tin chloride (SnCl_2_) (189.62 g/mol, Sigma-Aldrich, USA) were used as precursors. Ga(NO_3_)_3_·xH_2_O (0.5 M) and SnCl_2_ (0.5 M) were dissolved in 2-methoxyethanol(CH_3_OCH_2_CHOH) (Sigma-Aldrich, USA) as solvent and stirred on a hot plate at 50 °C for 6 h using a magnetic bar. A thermogravimetric analyzer (TGA) (N-1000, Sinco, Seoul, Korea) was used to investigate the thermal decomposition characteristics of the precursor sols. This measurement was carried out in a nitrogen ambient at a scanning rate of 10 °C/min. Figure 1 shows the TGA curves of Ga oxide, Sn oxide, and Ga:Sn oxide sols; the Ga:Sn oxide sol was prepared by mixing Ga oxide and Sn oxide precursor sols and its composition ratio was 1.0:1.0 in weight. The initial weight loss below 100 °C was due to the evaporation of the 2-methoxyethanol solvent, and the decomposition of organic compounds occurred. It is noted that the ligand exchange begins between the precursor and 2-methoxyethanol at low temperatures, and condensation occurs to form a partial network of metal-oxide bonds at elevated temperatures [16]. This led to a gradual decrease in weight with increasing temperature. Subsequently, the precursor sols exhibited negligible weight loss at temperatures above 500 °C. The difference in the remaining weights might be because the weight fractions of Ga and Sn atoms are different in each precursor solution; the weight percentage of the Ga atom in a gallium nitrate hydrate molecule is approximately 27.2 wt % and that of the Sn atom in a tin chloride molecule is approximately 62.6 wt %. A thermal annealing process at 500 °C was thus adopted to form oxide films from the prepared precursor solutions.

For the fabrication of TFTs, a *p*-doped silicon substrate with a 100-nm-thick silicon dioxide (SiO_2_) dielectric layer (LG Siltron, Gyeongsangbuk-do, Korea) was sequentially cleaned by ultrasonication in acetone, isopropyl alcohol, and deionized water. The substrate was subsequently treated with oxygen plasma for 10 s, while a radio frequency power of 40 W was applied and the oxygen flow rate was maintained at 9 sccm. Here, we remark that the oxygen plasma treatment is effective in removing undesirable organic contaminants, and this process is indispensable to generate a hydrophilic surface for interface suitability between the SiO_2_ dielectric and oxide semiconductor layers. To produce an oxide semiconductor layer, 0.5 M Ga oxide, 0.5 M Sn oxide, and Ga:Sn oxide precursor sols were used. In the Ga:Sn oxide precursor sols, the weight ratio of the Ga oxide and Sn oxide precursor sols, noted as a Ga:Sn composition, was varied as 1.0:0.5, 1.0:0.7, 1.0:0.9, 1.0:1.1, 1.0:1.3, and 1.0:1.5; the ratios of the number of Sn atoms to the number of Ga atoms in these Ga:Sn oxide sols were 0.85, 1.19, 1.53, 1.87, 2.21, and 2.56. Each precursor sol was filtered through a 0.2 μm PTFE filter and was then spin-coated on the pre-cleaned substrate at 4000 rpm for 35 s. The coated film was dried on a hot plate at 110 °C for 2 min to evaporate the solvent and then thermally annealed in a tube furnace at 500 °C for 1 h; these thermal treatments were performed in an ambient-air environment. Finally, 100-nm-thick Al source and drain electrodes were thermally deposited on the semiconductor layer through a shadow mask under a base pressure of 6 × 10^−6^ Torr. The channel length (L) and width (W) of the fabricated TFTs were 50 μm and 800 μm, respectively.

The chemical characteristics of the oxide films were investigated using an X-ray photoelectron spectroscope (K-Alpha, Thermo Scientific, Waltham, MA, USA), and the crystallographic properties were characterized using an X-ray diffractometer (DMAX-2500, Rigaku, Tokyo, Japan). The surface morphologies of the films were examined using a field-emission scanning electron microscope (S-4300, Hitachi, Ibaraki, Japan). A Hall effect measurement system (HMS-3000, ECOPIA, Anyang, Korea) was used to evaluate the electrical properties of the films. The electrical characteristics of the TFTs were evaluated using a semiconductor analyzer (4200-SCS, Keithley, Seoul, Korea).

## 3. Results and Discussion

**Figure 2:**
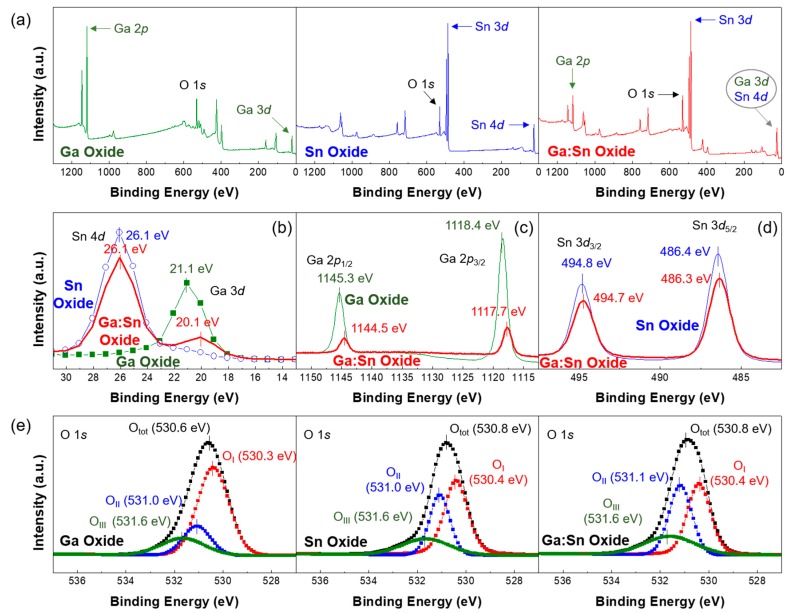
(**a**) XPS (X-ray photoemission spectroscopy) survey peaks of solution-processed Ga oxide, Sn oxide, and Ga:Sn oxide films; High-resolution XPS spectra of (**b**) Ga 3*d* and Sn 4*d* orbitals; (**c**) Ga 2*p* orbitals; and (**d**) Sn 3*d* orbitals; (**e**) O 1*s* XPS spectra in the Ga oxide, Sn oxide, and Ga:Sn oxide films.

XPS measurements were performed to quantitatively analyze the chemical elements and chemical valence of the Ga oxide, Sn oxide, and Ga:Sn oxide films. Figure 2a shows the XPS survey peaks of the solution-processed Ga oxide, Sn oxide, and Ga:Sn (composition ratio = 1.0:1.0) oxide films. In accordance with the literature, XPS peaks corresponding to Ga, Sn, and O are observed [17,18]. Therefore, it was confirmed that the Ga oxide, Sn oxide, and Ga:Sn oxide thin films were successfully formed in this study. In detail, the high-resolution XPS spectra of Ga 3*d*, Sn 4*d*, Ga 2*p*, and Sn 3*d* orbitals are compared in Figure 2b–d. Figure 2b shows that the peaks of Ga 3*d* and Sn 4*d* orbitals are located at approximately 21 eV and 26 eV, respectively [19,20]. With the mixing of Ga and Sn in the Ga:Sn oxide film, the separation distance between the Ga 3*d* and Sn 4*d* orbitals becomes approximately 6 eV. This allows Sn to be used as a substitutional or penetrating dopant in the Ga:Sn oxide film. It was also evident from Figure 2c,d that the Ga 2*p* and Sn 3*d* orbitals exhibit largely similar peak positions after the mixing. These results reveal that the chemical properties of Ga and Sn elements were not notably affected by the mixing.

As shown in Figure 2e, the high-resolution O 1*s* spectra can be deconvoluted into three peaks by Gaussian fitting; the peak binding energies of approximately 530.4 eV, 531.0 eV, and 531.6 eV were used in this fitting. In our study, O_I_, O_II_, and O_III_ were assigned as the binding energies of the three separated peaks; Table 1 summarizes the deconvolution results. The O_I_ peak located at the lowest binding energy of each case is attributed to the lattice oxygen and is formed because of Ga–O and Sn–O chemical bonding. The O_II_ peak could have resulted from the oxygen deficiency inside of the film and the O_III_ peak at the highest binding energy of each case is related to oxygen or OH species, such as O_2_ and H_2_O, adsorbed on the surface [21,22]. Here, the area ratio of O_I_/O_tot_ for the Sn oxide film is higher than that for the Ga oxide film because the chemical bond formed between Sn and O ions is stronger than that formed between Ga and O ions. The lowest O_I_/O_tot_ ratio for the Ga:Sn oxide film may be indicative of the incomplete conversion of mixed precursor sols to form metal–oxide bonds. To investigate the change in oxygen deficiency in the Ga:Sn oxide film, the area ratios of O_II_/O_tot_ were analyzed; the oxygen deficiency group is known to act as a donor [23,24]. The ratios of the Ga oxide, Sn oxide, and Ga:Sn oxide films were approximately 0.18, 0.30, and 0.36, respectively. Notably, the ratio of oxygen deficiencies in the Ga:Sn oxide film increased. Because Sn behaves as a substitutional atom in the Ga–O matrix and the crystalline phase in the Ga–O matrix can thus be changed, oxygen deficiency states are thought to increase in the Ga:Sn oxide film. In addition, the area ratio of O_III_/O_tot_ in the Ga:Sn oxide film is slightly higher than that in the Sn oxide film. This indicates that oxygen species remained in the Ga:Sn oxide film. In our results, the origin of oxygen species could not be clearly clarified; however, further research on this issue will be important for the comprehensive understating of the characteristics of the solution-processed oxide films. The observed variations in the O 1*s* spectra of the fabricated oxide films may have had an influence on the electrical properties of TFTs.

**Figure 3:**
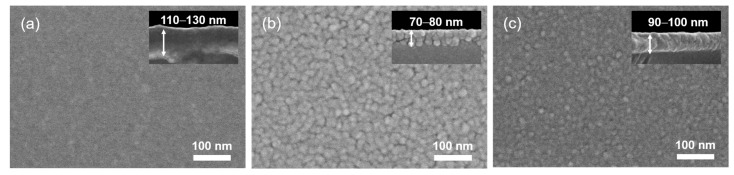
FE-SEM surface images of the (**a**) Ga oxide; (**b**) Sn oxide; and (**c**) Ga:Sn oxide films. The insets show the cross-sectional FE-SEM images of the films.

The surface morphologies of the solution-processed oxide films were observed via FE-SEM. Figure 3a shows an amorphous morphology of the fabricated Ga oxide film. Considering the ligand exchange and condensation processes to form the film from a precursor solution, the crystallization of solution-processed Ga oxide films, compared with vacuum-deposited films, may require higher annealing temperatures. Note that the substrate temperature critically affects the surface morphology of vacuum-deposited Ga oxide films and an amorphous phase is typically obtained when Ga oxide films are grown at low substrate temperatures below 400 °C [25]. The Sn oxide film exhibits a poly-grain, i.e., nanocrystalline state, as shown in Figure 3b. This indicates that solutes can be accommodated near the bulk and surface of the solution-deposited film, while the substrate heating causes the solidification to proceed from the bottom. In addition, grain coalescence and condensation on a film invariably occur during the spin-coating and annealing processes. These complexities eventually lead to the formation of grain boundaries in the solution-processed Sn oxide semiconductor film. In our results, several crack-type and void-type morphological defects are observed on the surface of the Sn oxide film, thereby degrading the packing density of grains in the film. Figure 3c shows the surface morphology of the Ga:Sn (composition ratio = 1.0:1.0) oxide film. Compared with the Sn oxide film, the Ga:Sn oxide film exhibits smaller grains. This demonstrates that the mixing of Ga with Sn affects the surface morphology of the film. From the cross-sectional FE-SEM images shown in the insets of Figure 3, the thicknesses of the Ga oxide, Sn oxide, and Ga:Sn oxide films were estimated to be 110–130 nm, 70–80 nm, and 90–100 nm, respectively.

**Figure 4:**
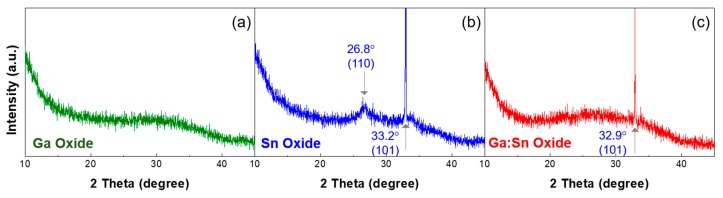
XRD (X-ray diffraction) patterns of the (**a**) Ga oxide; (**b**) Sn oxide; and (**c**) Ga:Sn oxide films, respectively.

To investigate the crystallinities of the solution-processed oxide films, the XRD in a 2*θ*-scan mode was used. Figure 4 show the XRD patterns of the Ga oxide, Sn oxide, and Ga:Sn (composition ratio = 1.0:1.0) oxide thin films. In Figure 4a, no diffraction peaks for the crystalline Ga oxide were observed in the Ga oxide film, indicating that the solution-processed Ga oxide film in this study is amorphous [26]. Figure 4b shows the presence of (110) and (101) peaks for the Sn oxide film, indicating the formation of a crystalline film [27]. In Figure 4c, the diffraction peaks for the crystalline Ga oxide are still not observed, while the (110) peak of Sn oxide disappeared. Accordingly, it is confirmed that the mixing of Ga with Sn inevitably causes the replacement of Ga^3+^ ions with Sn^4+^ ions. The morphological and structural properties are changed as Sn^4+^ ions substitute Ga^3+^ ions, and such structural transition is likely to induce more oxygen deficiencies in the Ga:Sn oxide films. 

**Figure 5:**
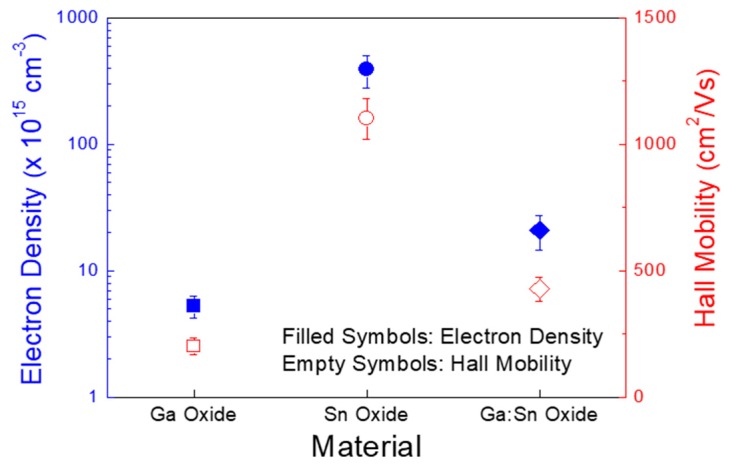
Electron concentrations and Hall mobilities of the Ga oxide, Sn oxide, and Ga:Sn oxide films.

Figure 5 shows the concentrations of electrons per unit volume and the Hall mobilities of the Ga oxide, Sn oxide, and Ga:Sn (composition ratio = 1.0:1.0) oxide films, which were measured using the Hall effect analyzer. Clearly, the Sn oxide film exhibited the largest electron concentration of approximately 3.9 × 10^17^ cm^−3^; those for the Ga oxide and Ga:Sn oxide films were approximately 5.2 × 10^15^ cm^−3^ and 2.1 × 10^16^ cm^−3^, respectively. Interestingly, though the oxygen deficiency of the Ga:Sn oxide film was observed to be the largest in Figure 2e, the electron concentration of the Ga:Sn oxide film was lower than that in the Sn oxide film in Figure 5. This may indicate a detrimental effect of oxygen species absorbed on the Ga:Sn oxide film. It is speculated that residual hydroxide groups in the Ga:Sn oxide film are likely to trap charges. Note that the area ratios of O_III_/O_tot_ of the Sn oxide and Ga:Sn oxide films were 0.41 and 0.51, respectively, and the hydroxide group is known to produce a charge trap state [28]. Compared to the Ga oxide film, the larger electron concentration of the Ga:Sn oxide film is thought to have originated from a larger concentration of oxygen deficiency, as shown in Figure 2e. In addition, the Sn oxide film also showed the highest Hall mobility of approximately 1102 cm^2^/Vs; those for the Ga oxide and Ga:Sn oxide films were approximately 201 and 428 cm^2^/Vs, respectively. We consider that the Hall mobilities of the fabricated films are closely associated with their crystallinities shown in Figure 4. Hence, the difference in the electron concentrations and the Hall mobilities of the solution-processed oxide films is critical for the electrical characteristics of TFTs.

**Figure 6:**
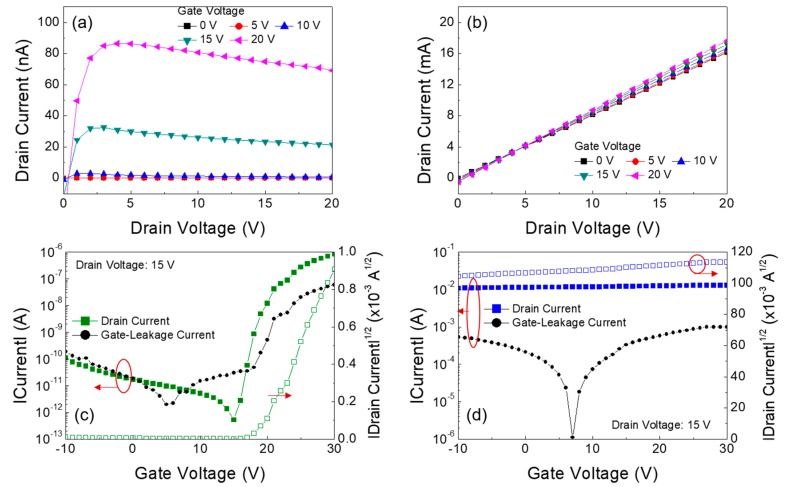
Output characteristics of the TFTs (thin-film transistors) fabricated with (**a**) Ga oxide and (**b**) Sn oxide films; Transfer characteristics of the TFTs fabricated with (**c**) Ga oxide and (**d**) Sn oxide films.

Figure 6a,b show the output characteristics of the Ga oxide and Sn oxide TFTs, respectively. These output characteristics were measured by changing the drain voltage (*V_D_*) from 0 to 20 V in increments of 1 V at different gate voltages (*V_G_*). The Ga oxide TFT exhibits a prime *n*-channel enhancement operation with pinch-off and saturation characteristics of the drain current (*I_D_*). The result indicates that electrons form the majority carriers in the Ga oxide semiconductor, whose transportation in the TFT is fundamentally controlled by positive gate and drain voltages. The negative drain currents for the Ga oxide TFT originated from the gate-leakage currents, which might be caused by the diffusion of Ga ions and/or atoms into the SiO_2_ dielectric layer. However, in the case of the Sn oxide TFT, the output currents are considerably high in the order of several milliamperes, which are not even amplified by the applied gate voltages. Figure 6c,d show the transfer characteristics of the Ga oxide and Sn oxide TFTs, respectively. These transfer characteristics were measured at a fixed drain voltage of 15 V, while the gate voltage was swept from −10 to 30 V in increments of 1 V. To evaluate the TFT performance, the subthreshold swing was defined as the change in the gate voltage required to change the drain current by a factor of 10, and the threshold voltage (*V_T_*) was extracted from the plot of |drain current|^1/2^ versus gate voltage by extrapolating to the drain current at 0 A. The field-effect mobility (*μ_eff_*) in the saturation region was calculated using the following relationship:

$$I_{D}=\frac{WC_{dielectric}}{2L}\mu_{eff}{\left(V_{G}-V_{T}\right)}^{2}$$

Here, *C_dielectric_* represents the capacitance of the gate dielectric layer. In our study, the Ga oxide TFT exhibited a field-effect mobility of 0.04 cm^2^/Vs, a subthreshold swing of 0.74 V/decade, a threshold voltage of 18 V, and a current on/off ratio of approximately 10^6^. The calculated field-effect mobility, which is lower than the Hall mobility shown in Figure 5, may be attributed to the impact of contact resistance, bulk resistance, channel resistance, and the semiconductor/dielectric interface characteristics on the charge transport behavior in the TFT structure. However, no switching function was observed for the Sn oxide TFT, as shown in Figure 6d. Here, the significant point is that the gate-leakage currents of the Sn oxide TFT is much higher than those of the Ga oxide TFT. This suggests that the diffusion of Sn ions into the SiO_2_ dielectric layer may be more severe than that of Ga ions. Previous studies reported the weak diffusion of Ga into SiO_2_ and the Arrhenius-type behavior of Sn diffusion into SiO_2_ [29,30,31]. The diffusivity of Sn into the SiO_2_ layer increases with temperature and is further augmented in the presence of H_2_O molecules, and diffused Sn atoms also extend to a larger depth inside the SiO_2_ layer. Note that the diffusion of metal ions and/or atoms into the dielectric layer of TFTs provides a current leakage path between source/drain and gate electrodes, thereby causing critically high levels of drain currents in the off-state region, which may explain the negative drain currents in the output characteristics of the Ga oxide TFT. Analyses on the depth profiles of Ga and Sn elemental distributions in the solution-processed oxide film and SiO_2_ dielectric layer can be conducted in future studies. In addition, the electrical properties exhibited by the Sn oxide, i.e., the largest electron concentration and the highest Hall mobility shown in Figure 5, are thought to cause a current conduction between the source and drain electrodes on the surface of the Sn oxide film. This is exemplified by the fact that drain currents were higher than gate-leakage currents and were not controlled by the gate voltage, as shown in Figure 6d. Therefore, the reason that the fabricated Sn oxide TFT exhibited no switching function can be understood by the diffusion of Sn ions into the SiO_2_ dielectric layer and the electrical properties of the Sn oxide film.

**Figure 7:**
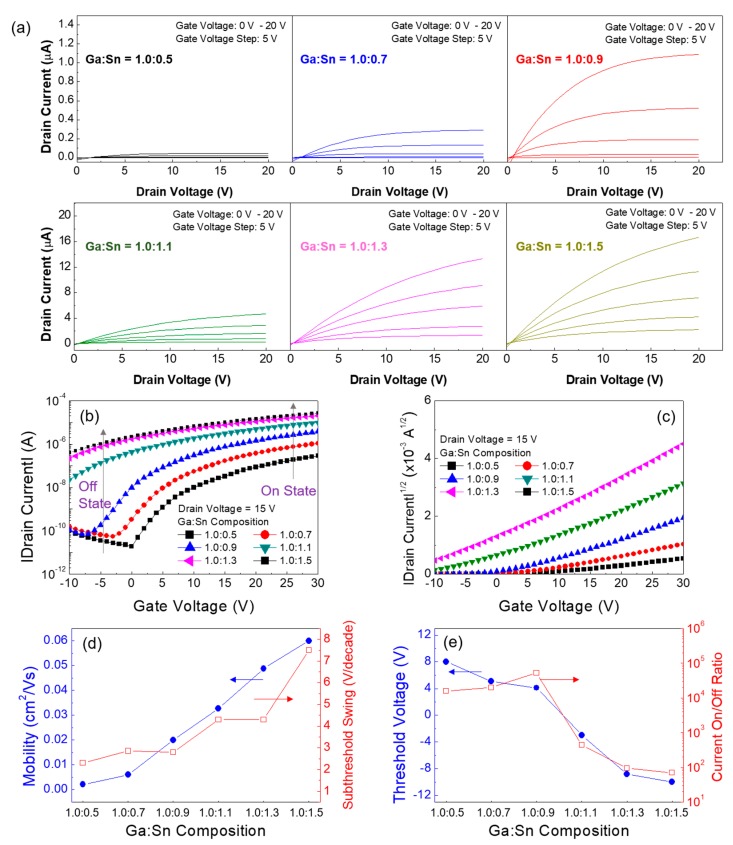
(**a**) Output characteristics of the Ga:Sn oxide TFTs. Transfer characteristic plots of (**b**) |drain current| versus gate voltage and (**c**) |drain current|^1/2^ versus gate voltage. Variations in the TFT characteristics according to the Ga:Sn composition: (**d**) field-effect mobility and subthreshold swing versus Ga:Sn composition, and (**e**) threshold voltage and current on/off ratio versus Ga:Sn composition.

Based on the results shown in Figure 6, the weight ratios of the Ga oxide and Sn oxide precursor sols were varied to investigate the effect of the composition ratio of Ga to Sn on the performance of Ga:Sn oxide TFTs. The molarity of each precursor solution was 0.5 M, whereas the weight ratios of the Ga oxide and Sn oxide precursor sols were changed (Ga:Sn composition = 1.0:0.5, 0.7, 0.9, 1.1, and 1.5). From the output characteristics shown in Figure 7a, the TFTs having different Ga:Sn compositions exhibit *n*-channel enhancement operation with pinch-off and saturation characteristics of the drain current. In particular, the drain current increases as Sn increases in the Ga:Sn oxide film. Figure 7b,c show the transfer characteristics of the fabricated Ga:Sn oxide TFTs. It can be clearly observed that, owing to the electrical properties of Sn, the drain currents in both the off-state and on-state regions increase as Sn increases in the Ga:Sn oxide film. Important TFT parameters extracted from the transfer characteristics are summarized in Figure 7d,e. As the Sn increased, the field-effect mobility increased and the threshold voltage decreased. These enhancements can be ascribed to an increase in electron concentration in the Ga:Sn oxide films as well as the electrical properties of Sn. Nevertheless, the subthreshold swing increased and the current on/off ratio decreased, which was caused from an increase in the drain current in the off-state region, as indicated in Figure 7b. These results demonstrate that the performance of the solution-processed Ga oxide TFTs can be optimized by substituting Ga with Sn. In this study, the optimal composition ratio of Ga:Sn in the solution-processed Ga:Sn oxide semiconductor is suggested to be 1.0:0.9 by taking into account the switching and amplification functions of typical TFTs.

**Figure 8:**
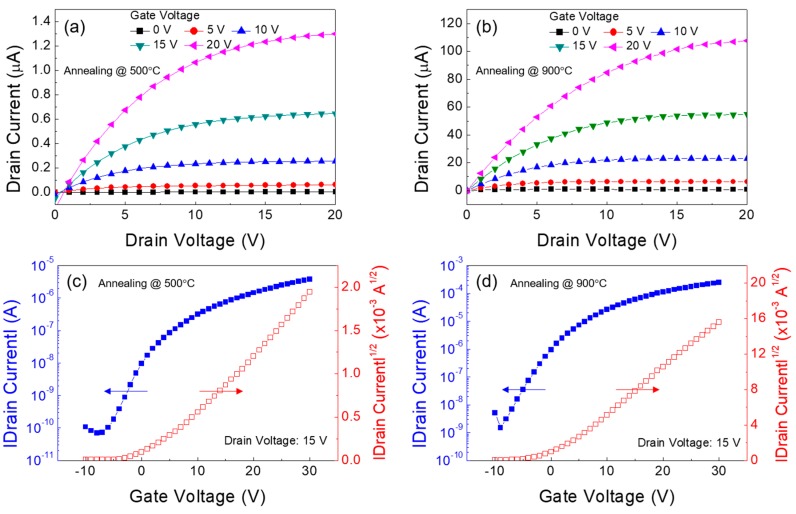
Output characteristics of the Ga:Sn oxide TFTs fabricated at the annealing temperature of (**a**) 500 °C and (**b**) 900 °C. Transfer characteristics of the TFTs fabricated at the annealing temperature of (**a**) 500 °C and (**b**) 900 °C.

**Figure 9:**
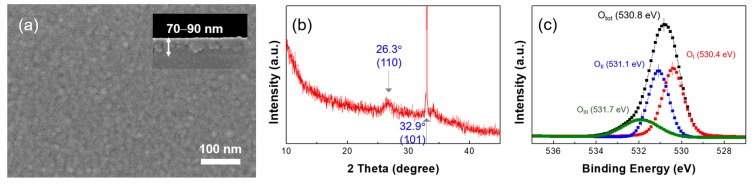
(**a**) FE-SEM images; (**b**) XRD pattern; and (**c**) O 1*s* XPS spectra of the Ga:Sn oxide film fabricated at the annealing temperature of 900 °C.

To further improve the TFT performance, the thermal annealing at a higher temperature of 900 °C for 1 h was conducted in a tube furnace for the formation of the Ga:Sn (composition ratio = 1.0:0.9) oxide film; this process was performed in an ambient-air environment. Figure 8a,c show the output and transfer characteristics of the TFT in which the Ga:Sn oxide film was thermally annealed at 500 °C for 1 h. The TFT exhibited a saturation drain current of approximately 1.3 μA (@ gate voltage = 20 V and drain voltage = 20 V), a field-effect mobility of 0.02 cm^2^/Vs, a subthreshold swing of 2.8 V/decade, a threshold voltage of 4.1 V, and a current on/off ratio of approximately 5.5 × 10^4^. In addition, the electrical characteristics of the Ga:Sn oxide TFT could be enhanced by thermally annealing the Ga:Sn oxide film at a higher temperature of 900 °C. From Figure 8b,d, a saturation drain current of approximately 107.6 mA (@ gate voltage = 20 V and drain voltage = 20 V), a field-effect mobility of 1.03 cm^2^/Vs, a subthreshold swing of 2.7 V/decade, a threshold voltage of 0.84 V, and a current on/off ratio of approximately 1.6 × 10^5^ were obtained. To elucidate these enhancements in the TFT performance, the characteristics of the solution-processed Ga:Sn oxide film annealed at 900 °C were investigated. From Figure 9a, this film exhibits a similar surface morphology with that of the Ga:Sn oxide film annealed at 500 °C; however, it is thinner (70–90 nm). In addition, as shown in Figure 9b, the XRD peak corresponding to the characteristic (110) peak of Sn oxide is observed, which disappeared in the film annealed at 500 °C. These results indicate that the crystallinity of solution-processed Ga:Sn oxide films can be enhanced as a consequence of thermal annealing at higher temperatures. By analyzing the O 1*s* spectrum of the Ga:Sn oxide film annealed at 900 °C shown in Figure 9c, it was found that the high-temperature annealing process resulted in an increase in the lattice oxygen (O_I_ peak) and a decrease in the oxygen species (O_III_ peak); the area ratios of O_I_/O_tot_, O_II_/O_tot_, and O_III_/O_tot_ were 45.1%, 37.6%, and 17.2%, respectively. The results of the Hall effect measurement also revealed an enhancement in the electrical properties of the Ga:Sn oxide film annealed at 900 °C, compared with the film annealed at 500 °C; the electron concentration and the Hall mobility were approximately 2.7 × 10^16^ cm^−3^ and 656 cm^2^/Vs, respectively. Consequently, we demonstrated that the mixing of Ga with Sn is viable for improving the performance of the solution-processed Ga oxide TFTs and the high-temperature annealing process is effective at enhancing the performance of the solution-processed Ga:Sn oxide TFTs. We expect high-pressure and UV-assisted annealing methods to be useful for lowering process temperatures to obtain high quality Ga:Sn oxide semiconductors in the future [32,33].

## 4. Conclusions

We investigated the effects of Ga and Sn compositions on the structural and chemical properties of Ga:Sn oxide films and the electrical properties of Ga:Sn oxide TFTs. From the results of the XPS analyses, the Ga:Sn oxide film exhibited a separation of approximately 6 eV between the binding energy positions of Ga 3*d* and Sn 4*d* orbitals, indicating that Sn atoms can be used as a substitutional or penetrating dopant in the Ga:Sn oxide film. In addition, it was found that the replacement of Ga ions with Sn ions led to an increase in oxygen deficiencies. In the SEM results, the Ga:Sn oxide film exhibited smaller grains compared to the nanocrystalline Sn oxide film, while the Ga oxide film exhibited an amorphous morphology. The XRD results revealed the change in the crystallinity of the Ga:Sn oxide film, compared to those of the Ga oxide and Sn oxide films. These structural characteristics also confirmed that the mixing of Ga with Sn causes the replacement of Ga ions with Sn ions. The results of the Hall effect measurement indicated that the increase in the oxygen deficiency ratio of the Ga:Sn oxide film contributed to an increase in electron concentration. For the Ga:Sn oxide TFTs, as the Sn content increased, the drain current and field-effect mobility increased and the threshold voltage decreased. However, when the content of the Sn oxide precursor sol increased to 1.1 (i.e., Ga:Sn composition = 1.0:1.1), the off-state current increased up to 10^−8^ A and the current on/off ratio eventually decreased to less than 10^3^. The electrical characteristics of the TFTs having different Ga:Sn oxide compositions can be explained through the increased electron concentration and the electrical properties of Sn. In our results, the optimal composition ratio of Ga:Sn in the solution-processed Ga:Sn oxide semiconductor was suggested to be 1.0:0.9. Furthermore, as the annealing temperature was increased to 900 °C, we found that the electrical characteristics of the Ga:Sn (1.0:0.9) oxide TFTs improved significantly. Therefore, we can also infer that the high-temperature annealing process contributes to the enhanced performance of the solution-processed Ga:Sn oxide TFTs. The results of this study are useful for analyzing the structural and electrical properties of solution-processed Ga:Sn oxide semiconductors in TFT applications. Further analyses on the depth profiles of Ga and Sn atoms in the vicinity of the interface between the Ga:Sn oxide semiconductor and SiO_2_ dielectric layers will serve to optimize the material composition and processing temperature for high performance Ga:Sn oxide TFTs.

## Figures and Tables

**Table 1:** Deconvolution results of O 1*s* XPS (X-ray photoemission spectroscopy) spectra for the fabricated oxide films.

Film	Area Ratio [%]		
	O_I_/O_tot_	O_II_/O_tot_	O_III_/O_tot_
Ga Oxide	64.6	18.5	16.9
Sn Oxide	50.1	30.7	19.2
Ga:Sn Oxide	41.6	36.8	21.6
